# The *Arabidopsis thaliana* Nuclear Factor Y Transcription Factors

**DOI:** 10.3389/fpls.2016.02045

**Published:** 2017-01-10

**Authors:** Hang Zhao, Di Wu, Fanying Kong, Ke Lin, Haishen Zhang, Gang Li

**Affiliations:** State Key Laboratory of Crop Biology, College of Life Sciences, Shandong Agricultural UniversityTai’an, China

**Keywords:** nuclear factor Y, *CCAAT* box, *Arabidopsis*, flowering time, hypocotyl elongation, abiotic response

## Abstract

Nuclear factor Y (NF-Y) is an evolutionarily conserved trimeric transcription factor complex present in nearly all eukaryotes. The heterotrimeric NF-Y complex consists of three subunits, NF-YA, NF-YB, and NF-YC, and binds to the *CCAAT* box in the promoter regions of its target genes to regulate their expression. Yeast and mammal genomes generally have single genes with multiple splicing isoforms that encode each NF-Y subunit. By contrast, plant genomes generally have multi-gene families encoding each subunit and these genes are differentially expressed in various tissues or stages. Therefore, different subunit combinations can lead to a wide variety of NF-Y complexes in various tissues, stages, and growth conditions, indicating the potentially diverse functions of this complex in plants. Indeed, many recent studies have proved that the NF-Y complex plays multiple essential roles in plant growth, development, and stress responses. In this review, we highlight recent progress on NF-Y in *Arabidopsis thaliana*, including NF-Y protein structure, heterotrimeric complex formation, and the molecular mechanism by which NF-Y regulates downstream target gene expression. We then focus on its biological functions and underlying molecular mechanisms. Finally, possible directions for future research on NF-Y are also presented.

## Main Text

Nuclear factor Y (NF-Y) is widespread in plants, animals, and other eukaryotes, and is also termed *CCAAT* Binding Factor (CBF) or Heme Activator Protein (HAP). The NF-Y complex consists of the subunits NF-YA (CBF-B/HAP2), NF-YB (CBF-A/HAP3), and NF-YC (CBF-C/HAP5), all of which are necessary for binding to the *CCAAT* box (Nardini et al., 2013). Individual subunits of NF-Y cannot regulate transcription independently; instead, they must function in heterodimers or heterotrimers. In yeast and mammals, each subunit of NF-Y is encoded by a single gene, but these genes have multiple splicing forms and undergo various post-translational modifications (Li et al., 1992a; Mantovani, 1999). In mammals, the physiological function and underlying molecular mechanism of the NF-Y complex has been extensively characterized in different cellular processes, such as endoplasmic reticulum stress, DNA damage, and cell cycle regulation (Oldfield et al., 2014; Benatti et al., 2016; Dolfini et al., 2016). In plants, each subunit of NF-Y is encoded by multiple members, which further form different sub-families (Petroni et al., 2012). A previous classification system described 36 NF-Y members (10 NF-YA, 13 NF-YB, and 13 NF-YC) in *Arabidopsis* (Siefers et al., 2009). This system considered the plant homologs of Negative Cofactors (NC2), DNA Polymerase II Subunit B3 (Dpb3), and Dpb4 as members of the NF-Y family; however, NC2, Dpb3p, and Dpb4p do not functionally overlap with NF-Y. NC2 associates with TATA-binding protein (TBP) to bind TATA boxes (Kamada et al., 2001), whereas Dpb3p and Dpb4p participate in the complex with DNA polymerase and the complex of chromatin remodeling (Ohya et al., 2000; Hartlepp et al., 2005). Therefore, the current consensus on *Arabidopsis* NF-Y members excludes AtNF-YC11/B12/B13 (NC2 subfamily) and AtNF-YC10/C13/B11 (Dpb3/4 subfamily), but does include AtNF-YC12. Thus, in the updated scheme, *Arabidopsis* has 30 members of the NF-Y family, 10 from each family (Petroni et al., 2012).

The initial reports of NF-Y genes in plants date back to the 1990s (Li et al., 1992b; Albani and Robert, 1995; Edwards et al., 1998). In the past decades, many studies have shed light on the biological functions of individual NF-Y subunits in *Arabidopsis* and other plant species, showing that this complex acts in gametogenesis, embryogenesis, seed development, flowering time regulation, primary root elongation, abscisic acid signaling, drought resistance, the endoplasmic reticulum stress response, hypocotyl elongation, and so on (Mantovani, 1999; Gusmaroli et al., 2001; Petroni et al., 2012 and references therein). All these studies suggest that the NF-Y gene family is a powerful and mysterious gene family and important for many aspects of plant life.

### The Protein Structure of NF-Y Subunits

**FIGURE 1:**
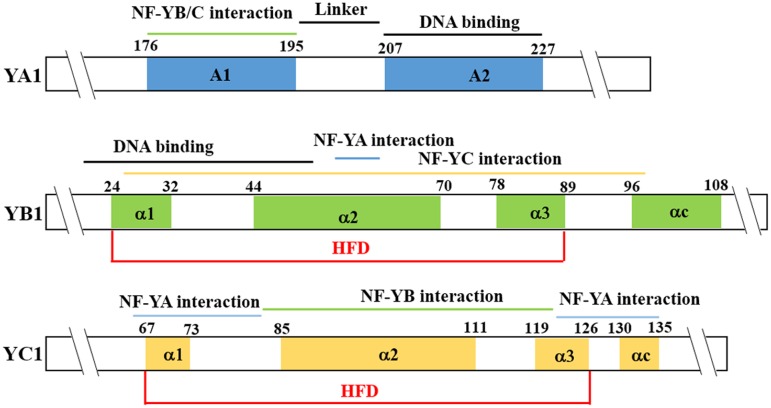
**Nuclear factor Y (NF-Y) protein structure**. The NF-YA conserved region is composed of two α-helices. The A1 helix is in the N-terminal of the core region and functions in interaction with NF-YB and NF-YC. The A2 is in the C-terminal and functions in specific recognition of the CCAAT element. The Histone Fold Domain (HFD) domain of the NF-YB and NF-YC proteins contains the DNA-binding domain and protein-protein interaction domain. The diagram shows AtNF-YA1, AtNF-YB1, and AtNF-YC1 as examples.

The A subunits of NF-Y generally localize to the nucleus and most NF-YA proteins can bind to the *CCAAT cis*-element in the promoter regions of target genes, but with different affinities (Calvenzani et al., 2012; Petroni et al., 2012; Laloum et al., 2013; Nardini et al., 2013). Protein structure analysis indicated that two conserved α-helix domains (A1 and A2) are present in the core regions of NF-YA subunits (**Figure 1**). The 20-amino-acid α helix A1 is in the N-terminal of the core region and functions in the interaction with NF-YB and NF-YC subunits, whereas the 21-amino-acid α helix A2 is in the C-terminal and provides sequence-specificity for recognition and binding of the *CCAAT cis*-element (Petroni et al., 2012; Laloum et al., 2013).

The NF-YB and NF-YC subunits contain the highly conserved Histone Fold Domain (HFD, also termed as Histone Fold Motif, HFM) (**Figure 1**), which functions in protein-DNA and protein-protein interactions (Frontini et al., 2004; Kahle et al., 2005; Laloum et al., 2013). The HFD domains of NF-YB and NF-YC subunits are more closely related to the core histone H2B and H2A, respectively (Dolfini et al., 2012; Petroni et al., 2012; Laloum et al., 2013). Typically, the HFD domain of NF-YB/YC is formed by a minimum of three α-helices (α1, α2, and α3) separated by two loops, similar to the HFD domains of histones H2B and H2A, and even similar to the HFD domain of the β and α subunits of NC2 (Arents and Moudrianakis, 1995; Mantovani, 1999). In NF-YB, the α1 helices contain the putative DNA-binding domain. In NF-YC, the α1 helices contain a few conserved amino acids which supposed to be the putative DNA-binding domains (Laloum et al., 2013). Indeed, the α1 helix makes essential stabilizing contacts with the DNA backbone flanking the central CCAAT pentamer (Nardini et al., 2013). NF-YB and NF-YC form a heterodimer via their HFDs, specifically via α2 and α3 (Kim et al., 1996; Zemzoumi et al., 1999; Frontini et al., 2004). The fourth helix of the conserved domain of NF-YB and NF-YC (called αC) has been proposed to function in interactions with other protein in mammals (Romier et al., 2003; Laloum et al., 2013). Formation of NF-YB/YC heterodimers is necessary for the translocation of NF-YB from the cytoplasm to the nucleus since NF-YB family members lack a nuclear localization signal (NLS), in contrast to the NF-YA and NF-YC subunits (Liu and Howell, 2010; Hackenberg et al., 2012). The HFD domains of NF-YB and NF-YC subunits assemble on a head-to-tail fashion to form a dimer, thus producing a structural scaffold for DNA-binding and interaction with NF-YA subunits (Nardini et al., 2013).

Crystal structure analysis of the NF-Y/CCAAT complex in mammals indicated that NF-YA binds to NF-YB/YC and inserts an α-helix into the minor groove of DNA, thus providing a sequence-specific contact to the *CCAAT* box. By contrast, NF-YB/NF-YC subunits form heterodimers that bind to the DNA sugar-phosphate backbone, mimicking the nucleosome H2A/H2B-DNA assembly (Romier et al., 2003; Nardini et al., 2013; Nardone et al., 2016). NF-YB/NF-YC interacts with DNA through non-specific HFD-DNA contact, which is similar to those of the core histone H2A/H2B. NF-YB/NF-YC also interact with NF-YA to produce the NF-Y complex with its sequence-specific recognition properties and nucleosome-like capabilities of non-specific DNA-binding. Thus combinations of subunits lead to stable DNA-binding activity of the NF-Y complex (Oldfield et al., 2014). Recently, the first crystal structure of the *Arabidopsis* NF-YB6/NF-YC3 dimer has been solved, which revealed that AtNF-YB6 and AtNF-YC3 subunits interact in a head-to-tail fashion and form a classical histone-like pair (Gnesutta et al., 2016; Nardone et al., 2016). The structural information on the NF-Y/CCAAT complex has given crucial insight into the molecular mechanism responsible for the architecture of the NF-YB/NF-YC heterodimer and the NF-Y heterotrimer, and also for the capacity of recognition and binding to DNA of NF-Y complexes.

### NF-Y Protein Complexes

There are 30 predicted NF-Y members in the *Arabidopsis* genome; in theory, this could result in about 1000 heterotrimeric combinations (Petroni et al., 2012). To define the molecular mechanism of NF-Y in plants, two fundamental questions about NF-Y complexes should be considered. First, how many heterotrimeric combinations actually exist *in vivo* and, second, what determines the specificity of those interactions and of DNA-binding. To define the unique NF-Y complexes, tissue- and development-specific expression patterns for all the subunits of *Arabidopsis* NF-Y have been investigated using stable promoter:beta-glucuronidase (GUS) fusion reporter lines (Siefers et al., 2009). The different subunits of NF-Y are specifically expressed in different tissues and organs during special developmental stages (Gusmaroli et al., 2001, 2002; Siefers et al., 2009; Cao et al., 2011; Sorin et al., 2014), or in response to environmental changes (Pant et al., 2009; Des Marais et al., 2012), suggesting that only some combinations of NF-Y subunits can be assembled and act in different developmental stages or under certain stimuli or conditions. In addition, yeast two-hybrid and three-hybrid systems have also been used to detect protein-protein interactions and formation of heterotrimers between *Arabidopsis* NF-Y subunits (Calvenzani et al., 2012; Hackenberg et al., 2012; Sato et al., 2014). Yeast two-hybrid assays indicated that most of the NF-Y subunits of the same type (A-A, B-B, or C-C) could not form homodimers or heterodimers, and NF-YA and NF-YB show little or no interaction. However, NF-YB and NF-YC subunits could interact in many combinations and form different heterodimers (Calvenzani et al., 2012; Hackenberg et al., 2012). These systematic studies of the protein interactions between NF-Y subunits have facilitated the discovery of complete NF-Y complexes, but tissue, development, and even time-dependent specific combinations should also be considered.

Research based on yeast three-hybrid and co-immunoprecipitation assays showed that NF-Y could function as a NF-YA-YB-YC heterotrimeric complex *in vivo* (**Table 1**) (Hou et al., 2014; Sato et al., 2014). In theory, *Arabidopsis* NF-Y subunits can form 1000 different heterotrimeric combinations (Petroni et al., 2012); however, only a few NF-Y complexes have been verified through molecular and biochemical studies (**Table 1**). One possible reason is that some or many heterotrimeric of NF-Y may be transient and highly dynamic *in vivo*, and thus hard to detect. Nevertheless, with the emergence of new experimental methods and in-depth studies of NF-Y family members, more and more NF-Y complexes are being identified and explored.

**Table 1:** Molecular function of different nuclear factor Y (NF-Y) protein complexes.

Protein complex	Molecular function	Reference
AtNF-YA1-B6-C10 AtNF-YA2-B2/5/6-C10 AtNF-YA4/7-B9-C10	Heat and drought response	Sato et al., 2014
AtNF-YA5-B9-C9	Blue light and abscisic acid responses.	Warpeha et al., 2007
AtNF-YA2-B10-C2	Unknown	Hackenberg et al., 2012
AtNF-YA4-B10-C2	Unknown	Hackenberg et al., 2012
AtNF-YA2-B2/B3-C9-CO-RGA	Regulate flowering time via regulating the expression of *SOC1/FT*	Hou et al., 2014; Xu F. et al., 2016
AtNF-YA4-B3-C2-bZIP28	Regulate ER stress	Liu and Howell, 2010
AtNF-YA2-B3-C10-DREB2A	Regulate heat tolerance via regulating the expression of *HsfA3*	Sato et al., 2014
AtNF-YB2, B3-C3, C4, C9-CO	Regulate flowering time via regulating the expression of *FT*	Kumimoto et al., 2010
AtNF-YB9-C2-bZIP67	Regulate seed development via activating expression of *CRC*	Yamamoto et al., 2009
AtNF-YB9-PIF4	Regulate hypocotyl elongation via regulating the expression of *IAA19*	Huang et al., 2015b
AtNF-YA2-RGA	Regulate flowering time	Hou et al., 2014
AtNF-YC1-CO	Regulate flowering time	Wenkel et al., 2006
AtNF-YC3-RGA	Regulate flowering time	Hou et al., 2014
AtNF-YC9-RGL2	Regulate seed germination	Liu et al., 2016

In the past few years, a growing number of studies have reported that subunits of NF-Y not only form heterodimeric and heterotrimeric complexes, but also interact with other proteins in various kinds of complexes. For example, multiple NF-YC subunits (C1, C3, C4, and C9) and NF-YB subunits (B2 and B3) can interact with CONSTANS (CO) to regulate flowering time (Wenkel et al., 2006; Kumimoto et al., 2010; Cao et al., 2014; Xu F. et al., 2016). In addition, basic region/leucine zipper motif (bZIP) type transcription factors bZIP28 and bZIP67 interact with NF-Y subunits to regulate endoplasmic reticulum (ER) stress and seed development, respectively (Yamamoto et al., 2009; Liu and Howell, 2010). Phytochrome interacting factor 4 (PIF4) interacts with LEAFY COTYLEDON1 (LEC1/NF-YB9) to regulate hypocotyl elongation (Huang et al., 2015b). REPRESSOR OF *ga1-3* (RGA) and RGA-LIKE2 (RGL2) interact with NF-YA2, NF-YB2/B3, or NF-YC3/C4/C9 to regulate gibberellin (GA) and photoperiod-mediated flowering time, or abscisic acid (ABA) and GA signaling pathways during seed germination, respectively (Hou et al., 2014; Liu et al., 2016). Studies of the interaction of NF-Y with other proteins regulating target gene expression have largely improved our understanding of the physiological roles of NF-Y in many biological activities (**Table 1**).

### NF-Y Regulates the Expression of Downstream Target Genes

**FIGURE 2:**
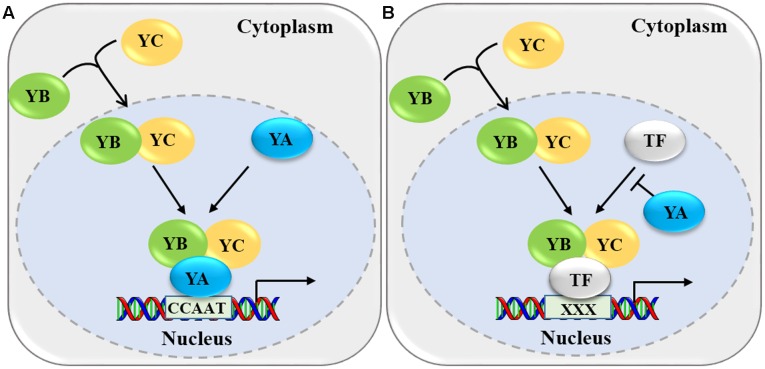
**Two molecular mechanisms by which NF-Y modulates gene expression. (A)** The NF-YB-YC heterodimers translocate into the nucleus and interact with NF-YA to form an active heterotrimer. This heterotrimer binds to the CCAAT box in the promoter regions of the target genes and mediates their expression. **(B)** The NF-YB-YC heterodimer interacts with the transcription factor (TF) to form a NF-YB-YC-TF heterotrimer and binds to specific *cis*-element (indicated by XXX). NF-YA can competitively inhibit the interaction of the TF with NF-YB/NF-YC.

Since NF-Y regulates gene expression mainly as a protein complex, it is important to understand its specific regulatory mechanisms. Previous reports have proposed that NF-Y modulates expression of downstream target genes mainly by two mechanisms (**Figure 2**). In the first mechanism, the NF-YB-YC heterodimer assembles in the cytoplasm, then translocates into the nucleus, where it interacts with NF-YA to form an active heterotrimer (**Figure 2A**) (Hackenberg et al., 2012; Laloum et al., 2013). The NF-YA-YB-YC complex mainly binds to the *CCAAT* box in the promoter regions of the downstream target genes through NF-YA, and regulates the expression of target genes, which is a highly conserved transcriptional regulation mechanism for NF-Y complexes in yeast, mammals, and plants (Mantovani, 1999; Frontini et al., 2004; Liu and Howell, 2010; Dolfini et al., 2012; Petroni et al., 2012; Hou et al., 2014; Sato et al., 2014). For example, the AtNF-YA4-YC2-YB3 complex binds to the *CCAAT cis*-element in the promoter region of *BINDING PROTEIN 3* (*BiP3*) through AtNF-YA4 and up-regulates the expression of ER stress-induced genes (Liu and Howell, 2010). In addition, the AtNF-YA6 subunit interacts with the AtNF-YB6/YC3 dimer and the resulting trimer can directly bind *CCAAT cis*-elements, even a probe containing the *CCAAT* box derived from the human *Heat Shock Protein70* (*HSP70*) promoter (Nardone et al., 2016). Most NF-Y complexes bind to the *CCAAT* box mainly through NF-YA; however, in plants, a recent study in rice reported that OsNF-YB1 might bind to the *CCAAT* box of *Sucrose Transporter1* (*SUT1*), *SUT3*, and *SUT4* (Bai et al., 2016). However, the *in vitro* and *in vivo* data are not sufficient to prove OsNF-YB1 binding to *CCAAT* box directly, since some sequences flanking the *CCAAT* box could bind via NF-YB (Nardini et al., 2013).

In the second molecular mechanism, the NF-YB/NF-YC heterodimer associates with specific transcription factors to form a complex (NF-YB-YC-transcription factor), and regulates the expression of their target genes through the binding of transcription factors to specific *cis*-elements in the promoters of various target genes (Wenkel et al., 2006; Yamamoto et al., 2009; Kumimoto et al., 2010) (**Figure 2B**). In this mechanism, NF-YA probably could suppress the formation of the NF-YB-YC-transcription factor trimeric complex by preventing the combination of transcription factors with NF-YB-YC. Although this kind of transcriptional regulation has been proposed by many studies, while the direct molecular evidences are still required since the opinion of compete of YA with TF derived from supposes upon some opposite observations. For example, AtB9-C2-bZIP67 directly binds to the ABA-response elements (ABREs) through bZIP67 in the promoter regions of *SUCROSE SYNTHASE 2* (*SUS2*) and *CRUCIFERIN C* (*CRC*), then activates the expression of *SUS2* and *CRC* and promotes seed development (Yamamoto et al., 2009). However, NF-YA subunits strongly inhibited the expression of *CRC* by competing with bZIP67 to form an NF-YA-YB9-YC2 complex, which indicated different members of the NF-Y subunits play distinct roles in plant development and growth (Adrian et al., 2010). In mammals, genome-wide association with selected transcription factors indicated that the NF-Y complex directly binds to the *CCAAT* box and associates with other *cis*-elements by interacting with different transcription factors (Dolfini et al., 2016; Zambelli and Pavesi, 2016). Recently, a ChIP-sequencing approach in rice identified genome-wide downstream targets of OsNF-YB1, indicating that OsNF-YB1 directly binds to the *CCAAT* box and associates with the *GCC* box by interacting with an ERF-type transcription factor (Xu J.J. et al., 2016).

Some special transcriptional regulatory mechanisms do not fit these two major regulatory models in *Arabidopsis*. For instance, AtNF-YA2 can directly bind to the *NFYBE cis*-element (not the *CCAAT* box) of the *SUPPRESOR OF OVEREXPRESSION OF CONSTANS1* (*SOC1*) promoter to regulate the expression of *SOC1* (Hou et al., 2014). In addition, a recent study indicated that NF-YC subunits (C1, C3, C4, and C9) directly interact with HISTONE DEACETYLASE 15 (HDA15), recognize the promoter of hypocotyl elongation-related genes, and repress their expression through the deacetylation of H4 in a light-dependent manner (Tang et al., 2016). A previous study also showed that NF-Y directly activates the expression of *SOC1* partly through the H3K27 demethylase RELATIVE OF EARLY FLOWERING 6 (REF6; Hou et al., 2014). Therefore, the NF-Y complex may function together with various epigenetic factors to regulate the transcription of downstream target genes. Certainly, deeper research will discover more and more novel mechanisms.

### The Biological Functions of NF-Y Subunits

#### Embryo Development and Seed Germination

NF-Y transcription factors play crucial roles in embryogenesis. The first identified and extensively studied NF-Y subunit acting in embryogenesis and seed maturation is NF-YB9, which was identified as LEAFY COTYLEDON1 (LEC1; West et al., 1994; Lotan et al., 1998; Lee et al., 2003). NF-YB9/LEC1 plays multiple essential roles in embryogenesis and post-embryonic development in *Arabidopsis*, where it is required to maintain the fate of embryonic cells and prevent immature seeds from germinating prematurely (Meinke et al., 1994; West et al., 1994; Lotan et al., 1998; Lee et al., 2003; Suzuki et al., 2007; Yamamoto et al., 2009; Junker et al., 2012; Mu et al., 2013). *NF-YB9* and *LEC1-LIKE* (*L1L*/*NF-YB6*) affect embryo development through induction of genes related to embryogenesis and cellular differentiation (Lotan et al., 1998; Lee et al., 2003; Huang et al., 2015a).

Based on the tissue-specific expression patterns and mutant phenotypes, many *Arabidopsis* NF-Y genes participate in embryo development. For example, *NF-YA1, YA2, A3, A4, A6, A7, A8*, and *A9* are expressed in the embryo and may affect embryo development (Siriwardana et al., 2014). Moreover, the transgenic plants overexpressing *NF-YA1*, *A5*, *A6*, or *A9* show hypersensitivity to ABA during seed germination and promotion of the vegetative-to-embryonic transition. However, single or double mutants of the four *NF-YA* genes do not have detectable phenotypes (Mu et al., 2013). *NF-YA3* and *A8* show their highest expression in the embryo from the globular to torpedo stages. The *nf-ya3 nf-ya8* double mutants are embryo lethal, but the *nf-ya3* and *nf-ya8* single mutants do not display an obvious phenotype, indicating that *NF-YA3* and *NF-YA8* function redundantly in early embryogenesis of *Arabidopsis* (Fornari et al., 2013). Therefore, multiple individual NF-YA subunits likely play redundant roles in embryo development and seed germination.

Recent research indicates that NF-YC subunits are also involved in seed germination through ABA responses and different NF-YC subunits have both unique and opposing functions in ABA-mediated seed germination (Kumimoto et al., 2013). For example, *Arabidopsis nf-yc4* single mutants are hypersensitive to ABA during seed germination (Warpeha et al., 2007), but *nf-yc3* n*f-yc9* double mutants are hyposensitive to ABA (Kumimoto et al., 2013). Although extensive genetic evidence indicated that many individual NF-Y subunits function in embryonic and seed development, and seed germination, most studies focus on single subunits; therefore, how different subunits act in the NF-Y complexes to regulate these process remains elusive.

#### Hypocotyl Elongation under Light Signaling Pathway

NF-Y also functions in plant photomorphogenesis, especially in hypocotyl elongation (Leyva-González et al., 2012; Mu et al., 2013; Huang et al., 2015b; Myers et al., 2016). Previous genetic evidence showed that overexpression of *AtNF-YA1* and *NF-YA9* changed cell identity, and led to the hypocotyl becoming greener and swollen (Mu et al., 2013). In addition, overexpression of most NF-Y A subunits significantly reduced hypocotyl elongation (Leyva-González et al., 2012; Myers et al., 2016). However, the underlying molecular mechanism by which different A-type subunits of NF-Y regulate hypocotyl elongation requires further investigation.

**FIGURE 3:**
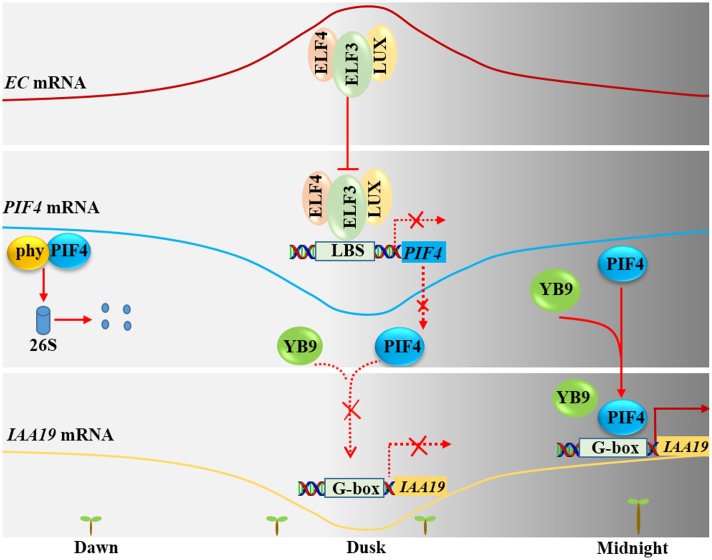
**Nuclear factor Y regulates hypocotyl elongation through PIF4**. At dawn, Phytochrome interacting factor 4 (PIF4) is degraded by the 26S proteasome. At dusk, the transcription level of *PIF4* is repressed by evening complex (ELF4-ELF3-LUX). Therefore, NF-YB9/LEC1 cannot be recruited to the promoter of *IAA19*. At midnight, the transcription level of *PIF4* increases along with decreased abundance of the ELF4-ELF3-LUX complex. Meanwhile, NF-YB9/LEC1 is recruited to the promoter of *IAA19* through its interaction with PIF4, thus increasing the transcription level of *IAA19* and promotes hypocotyl elongation.

After seed imbibition, induced expression of *NF-YB9/LEC1* resulted in longer hypocotyls, compared with non-induced seedlings, and mutants of *NF-YB9* had short hypocotyls (Junker et al., 2012; Huang et al., 2015b). In addition, genome-wide chromatin immunoprecipitation (ChIP) microarray (ChIP-chip) analysis revealed that a number of auxin and cell elongation-related genes (such as *YUC10*, *IAA5*, and *IAA19*) are putative target genes of NF-YB9/LEC1 (Junker et al., 2012). Moreover, previous studies reported that the evening complex (EC, ELF4-ELF3-LUX) binds to the promoter regions of *PIF4*/*PIF5* through the transcription factor LUX to repress the expression of *PIF4* and *PIF5* in the evening (Nusinow et al., 2011). Further study found that PIF4 could interact with NF-YB9 to coordinately regulate the expression of *IAA19* and thus control the elongation of the hypocotyl in the dark (**Figure 3**) (Huang et al., 2015b). At dawn, PIF4 protein interacts with photoactivated phytochrome and then is degraded through the 26S proteasome pathway. With prolonged illumination, the ELF4-ELF3-LUX complex accumulates, and reaches a peak at dusk. The accumulated ELF4-ELF3-LUX complex can repress the transcription of *PIF4*, thus suppressing the binding of the PIF4-NF-YB9 complex to the promoter region of *IAA19* and inhibiting its expression. Therefore, *Arabidopsis* hypocotyl elongation is inhibited at this stage. At midnight, the levels of the ELF4-ELF3-LUX EC decrease, allowing the restoration of *PIF4* mRNA levels and accumulation of PIF4 protein. So, PIF4 can recruit NF-YB9 to form a complex and target the promoter region of *IAA19*, positively regulating *IAA19* expression. Thus, *Arabidopsis* hypocotyl elongation gradually increases at this stage (**Figure 3**).

Recent reports demonstrated that five NF-YC subunits (C1, C3, C4, C6, and C9) also function in hypocotyl elongation by interacting with HDA15 (Myers et al., 2016; Tang et al., 2016). The triple mutant of *nf-yc3 nf-yc4 nf-yc9* displayed longer hypocotyls under blue, and red light conditions; therefore these NF-YC subunits positively regulate photomorphogenesis (Myers et al., 2016; Tang et al., 2016). Although all three types of NF-Y subunits function in hypocotyl elongation, the underlying molecular mechanism by which NF-Y complexes regulate photomorphogenesis and hypocotyl elongation remains elusive.

#### Flowering Time Regulation

**FIGURE 4:**
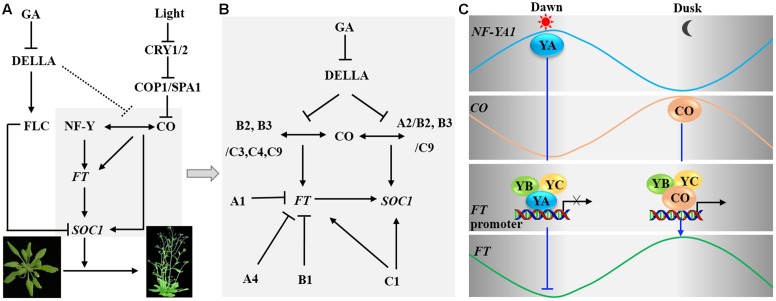
**Nuclear factor Y controls flowering time. (A)** In the photoperiod pathway, the NF-Y complex can interact with CONSTANS (CO) protein, then binds the promoter region of *FT* and enhances its transcription. In the gibberellin (GA) signaling pathway, DELLA interacts with FLC and enhances transcriptional repression of *FT* and *SUPPRESOR OF OVEREXPRESSION OF CONSTANS1 (SOC1)* by FLC. DELLA also represses the interaction of CO with NF-YB2, thus preventing the CO-NF-Y complex from promoting expression of *FT* and *SOC1*. **(B)** A diagram indicating the detailed molecular mechanism is shown in the region in gray in **(A)**. NF-YA1, YA4, and YB1 repress the expression of *FT* and thus delay flowering; NF-YC1 increases the transcription of *FT* and *SOC1*, thus promoting flowering. NF-YB2/B3 and YC3/YC4/YC9 can form a complex with CO and regulate the expression of *FT*. Moreover, NF-YA2/YB2 and NF-YB3/YC9 interact with CO and regulate the expression of *SOC1*. **(C)** CO and NF-Y regulate *FT* expression. At dawn, NF-YA1-YB-YC form a heterotrimeric complex that binds to the promoter of *FT* thus repressing its transcription. At dusk, CO competes with NF-YA1 to form a heterotrimeric complex NF-YB-YC-CO to promote the transcription of *FT*.

The timing of the transition from vegetative to reproductive development is crucial for reproductive success of flowering plants. In *Arabidopsis*, the major flowering pathways include photoperiod and GA pathways, which promote flowering in response to seasonal changes in day length and the endogenous content of GA, respectively. The interactions of these flowering pathways regulate the expression of two floral pathway integrators, *FT* and *SOC1*, which in turn activate the genes involved in the formation of floral meristems (Brambilla and Fornara, 2016). Overexpression of many individual NF-Y subunits (such as *NF-YA1*, *YA4*, *NF-YB1*, *B2*, *B3*, *NF-YC1*, *C2*, *C3*, *C4*, and *C9*) altered flowering time, indicating that NF-Y complexes regulate flowering time by a highly redundant and complicated mechanism. Multiple individual subunits of NF-Y can interact with CO and affect the transcript levels of *FT* and *SOC1*, two key integrators in the flowering time pathway, thus resulting in early or late flowering (**Figure 4A**) (Kumimoto et al., 2010; Cao et al., 2014; Hou et al., 2014; Xu F. et al., 2016).

In the photoperiod-dependent pathway, AtNF-YB2 and B3 have additive roles in the positive regulation of *FT* expression and flowering time (Kumimoto et al., 2008). Further studies indicate that the NF-YC subunits AtNF-YC3, C4, and C9 interact with AtNF-YB2, B3, and CO to form NF-YB-YC-CO complexes, and NF-YB and NF-YC subunits are required for the transcriptional activation of *FT* mediated by CO under long-day (LD) conditions (Kumimoto et al., 2010) (**Figure 4B**). In addition, two *CO-Responsive Elements* (*CORE1* and *2*) present in the proximal region and the *CCAAT* box present in the distal region of the *FT* promoter are crucial for regulation of *FT* expression (Adrian et al., 2010; Tiwari et al., 2010; Cao et al., 2014). CO directly binds to *CORE2*, interacts with NF-Y complexes, which can bind to the *CCAAT* box, resulting in chromatin loops, and positively regulates the expression of *FT* (Tiwari et al., 2010; Cao et al., 2014). Whether CO associates with the *CORE2* in the NF-YB-YC-CO complex and how protein binding to the *CORE* can drive *FT* expression remain to be investigated (Adrian et al., 2010). Conversely, some NF-Y subunits can delay flowering by repressing the transcription of *FT*. For example, the overexpression of *AtNF-YA1*, *A4*, and *AtNF-YB1* reduced the transcript level of *FT* and postponed flowering (Wenkel et al., 2006; Nelson et al., 2007). Further studies have shown that NF-YA might delay flowering by preventing the interaction between CO and NF-YB/NF-YC. For instance, overexpression of *NF-YA1* and *A4* prevents the interaction between CO and the NF-YB/NF-YC heterodimer, finally reducing the expression of *FT* and postponing flowering (Wenkel et al., 2006; Nelson et al., 2007; Leyva-González et al., 2012). Thus, different subunits of NF-Y might act in different complexes and have opposite effects on the final phenotypic output.

In the regulation of flowering time, both the NF-YB-YC-CO complexes and NF-YA-YB-YC complexes can bind to *FT* promoter (Adrian et al., 2010; Cao et al., 2014; Brambilla and Fornara, 2016). Indeed, both the transcript and protein levels of CO and NF-Y undergo rhythmic changes during the day; thus the complexes of NF-YA-YB-YC and the complexes of NF-YB-YC-CO likely change dynamically over time (**Figure 4C**). The protein level of CO decreases gradually from night to dawn (Song et al., 2015). Meanwhile, increasing NF-YA1-YB-YC forms a trimer and binds to the *FT* promoter to repress *FT* expression (Wenkel et al., 2006). In addition, CO protein accumulates gradually during the day and its expression peaks at dusk; thus CO can compete with NF-YA1 to form the trimeric complex NF-YB-YC-CO and promote the transcription of *FT* leading to peak expression at dusk (Wenkel et al., 2006).

NF-Y also affects flowering time by interacting with DELLA protein in the GA-dependent pathway (**Figure 4**). A study using ChIP showed that the NF-YA2/B2/C9 complex could interact with CO and DELLA and bind to the *NFYBE cis*-element in the promoter of *SOC1* through NF-YA2. The NF-Y complex then recruits REF6 to demethylate H3K27me3, thus promoting *SOC1* expression and leading to early flowering (Hou et al., 2014). Under LD conditions, RGA (a member of the DELLA protein family) not only interacts with NF-YB2 and CO but also represses the interaction of CO with NF-YB2. This interaction was tested through *in vitro* pull-down assays, but *in vivo* tests need further investigate (Hou et al., 2014). In addition, a previous study reported that NF-YB2 and CO form a complex with part of NF-YC family members to affect *FT* expression (Kumimoto et al., 2010). Therefore, the effect of RGA on CO-NF-Y binding to the promoter of *SOC1* very likely represses the activity of CO in activating *FT* expression (Xu F. et al., 2016).

#### Abiotic Stress

*Arabidopsis* NF-Y also has an important role in the responses to abiotic stresses, such as drought, salt, cold, and heat (**Table 2**). Many NF-YA, NF-YB, and NF-YC subunits play key roles in the drought response (Nelson et al., 2007; Li et al., 2008; Hackenberg et al., 2012). Overexpressing *NF-YA5* reduced leaf water loss and increased resistance to drought stress compared with the wild type, whereas *nf-ya5* mutants were more sensitive to drought stress, which suggests that NF-YA5 positively regulates drought stress responses (Li et al., 2008). Beside *NF-YA5*, overexpression of other *NF-YA* genes such as *YA2, A3, A7*, and *A10* also increased the tolerance to drought, which suggests that NF-Y subunits share overlapping functions in stress responses (Leyva-González et al., 2012). In addition to NF-YA, NF-YB is also involved in *Arabidopsis* drought stress tolerance. Overexpression of *NF-YB1* enhanced plant drought resistance (Nelson et al., 2007). However, this did not affect the expression of genes involved in ABA signaling, indicating that NF-YB1 might regulate drought stress responses independent of ABA signaling (Nelson et al., 2007).

**Table 2:** The functions of *NF-Y* genes in *Arabidopsis.*

Gene name	Other name	Biological function	Reference
*AtNF-YA1*	*AtHAP2a*	Flowering time regulation; salt stress response; seed morphology regulation; early embryogenesis.	Wenkel et al., 2006; Li et al., 2013; Mu et al., 2013
*AtNF-YA2*	*AtHAP2b*	Flowering time regulation; root growth.	Sorin et al., 2014; Hou et al., 2014
*AtNF-YA3*	*AtHAP2c*	Early embryogenesis; nitrogen nutrition.	Fornari et al., 2013
*AtNF-YA4*		Flowering time regulation; ER stress.	Liu and Howell, 2010; Leyva-González et al., 2012
*AtNF-YA5*		Drought resistance; early embryogenesis.	Warpeha et al., 2007; Li et al., 2008; Mu et al., 2013
*AtNF-YA6*		ABA response and seed germination; blue light signaling transduction; plant fertility.	Mu et al., 2013
*AtNF-YA8*		Early embryogenesis.	Fornari et al., 2013
*AtNF-YA9*		Embryogenesis; seed morphology regulation; seed germination.	Mu et al., 2013
*AtNF-YA10*		Root growth.	Sorin et al., 2014
*AtNF-YB1*	*AtHAP3a*	Flowering time regulation; drought resistance.	Wenkel et al., 2006; Nelson et al., 2007
*AtNF-YB2*	*AtHAP3b*	Root development; flowering time regulation.	Kumimoto et al., 2008; Ballif et al., 2011;Xu F. et al., 2016
*AtNF-YB3*		Flowering time regulation; ER stress; Heat stress response.	Liu and Howell, 2010; Sato et al., 2014
*AtNF-YB6*	*LEC1-L*	Early embryogenesis.	Lee et al., 2003
*AtNF-YB9*	*LEC1*	Early embryogenesis; hypocotyl elongation.	Warpeha et al., 2007; Junker et al., 2012; Huang et al., 2015b
*AtNF-YC1*	*AtHAP5a*	Flowering time regulation; cold tolerance	Hackenberg et al., 2012; Shi et al., 2014
*AtNF-YC2*	*AtHAP5b*	Flowering time regulation; ER stress; drought resistance.	Liu and Howell, 2010; Hackenberg et al., 2012
*AtNF-YC3*		Flowering time regulation.	Kumimoto et al., 2010
*AtNF-YC4*		Flowering time regulation; increasing protein and decreasing starch levels.	Kumimoto et al., 2010
*AtNF-YC9*	*AtHAP5c*	Flowering time regulation; chlorophyll biosynthesize.	Warpeha et al., 2007; Kumimoto et al., 2010; Hou et al., 2014
*AtNF-YC10*	*DPB3-1*	Heat stress response	Sato et al., 2014

A previous study demonstrated that overexpression of *NF-YC1* improved freezing resistance, whereas *nf-yc1* mutants exhibited decreased freezing resistance, which suggests that NF-YC1 positively regulates freezing responses (Shi et al., 2014). Further research found that NF-YC1 improved freezing resistance by binding to the CCAAT *cis*-elements in the promoter region of *Xyloglucan Endotransglucosylase/Hydrolase 21* (*XTH21*) in *Arabidopsis* (Shi et al., 2014). Further, overexpression of NF-YA1 enhanced plant resistance to salt stress by increasing the expression of *Abscisic Acid Insensitive3* (*ABI3*) and *Abscisic Acid Insensitive5* (*ABI5*; Li et al., 2013). In addition, the NF-YA2-B3-C10 ternary complex enhanced the expression of the heat stress-inducible gene *HEAT SHOCK FACTOR A3* (*HsfA3*) during heat stress responses in cooperation with DREB2A (Sato et al., 2014).

#### Other Functions of NF-Y

In addition to these above functions, NF-Y also plays roles in other plant processes. For example, NF-YA2, A3, and A5 participate in nitrogen nutrition (Zhao et al., 2011; Leyva-González et al., 2012); NF-YA2, NF-YA10, and NF-YB2 function in the control of primary root growth (Ballif et al., 2011; Sorin et al., 2014); NF-YB1 affects shoot apical meristem growth (Wenkel et al., 2006); NF-YC4 is involved in starch and protein metabolism (Li et al., 2015); and NF-YA5/B9/C9 associates with chlorophyll biosynthesis (Warpeha et al., 2007).

### Perspectives

In plant, the different individual subunits of NF-Y have attracted extensive attention in recent years, but the many functions of the NF-Y complex remain only partially defined. In contrast to yeast and mammals, plants have a large NF-Y family, generally having multi-gene families encoding each subunit. This provides more combinations of NF-YA-YB-YC complexes in different developmental stages or under certain conditions, and increases functional complexity. This indicates that NF-Y is widely involved in the intricate regulatory processes in plants, compared with its more narrow roles in other organisms.

The downstream target genes and upstream regulators of NF-Y, and its functional redundancy and specificity are largely unknown. The diversity of NF-Y subunits and their many potential combinations, as well as likely redundant and divergent functions, provide a substantial challenge for work aiming to tease apart the functions of the different subunits. In mammals, NF-Y is a pioneer factor that binds to the *CCAAT* box in the core promoter and enhancer region (Nardini et al., 2013; Oldfield et al., 2014), but its targets remain elusive in plants. In addition, other fundamental questions about NF-Y, such its transcriptional regulation, and post-translational modification, need to be investigated. NF-Y complexes definitely function as essential regulatory hubs for many processes in plant, but its functional redundancy remains a problem for further investigation. Therefore, future studies on NF-Y will play an indispensable part in plant science.

## Author Contributions

HZ and GL conceived the manuscript; HZ and DW drafted the manuscript. HZ, GL, FK, HZ, and KL edited the draft, and all authors approved the final version of manuscript.

## Conflict of Interest Statement

The authors declare that the research was conducted in the absence of any commercial or financial relationships that could be construed as a potential conflict of interest. The reviewer JPL and handling Editor declared their shared affiliation, and the handling Editor states that the process nevertheless met the standards of a fair and objective review.
